# Comparison of Polyethylene Wear before and after Hip Revision with Liner Exchange Fixed with the Original Locking Mechanism

**DOI:** 10.1371/journal.pone.0167607

**Published:** 2016-12-09

**Authors:** Xinfeng Gu, Jie He, Yiwen Tang, Yuxin Zheng

**Affiliations:** Department of Orthopedics and Traumatology, Shuguang Hospital Affiliated to Shanghai University of Tradition Chinese Medicine, Shanghai, China; Institute of Materials Research and Engineering, SINGAPORE

## Abstract

**Objective:**

To compare the wear of conventional ultra-high molecular weight polyethylene (CUHMWPE) and highly cross-linked polyethylene (HCLPE) in hip revision with liner exchange fixed with original locking mechanism using analysis of history medical data.

**Methods:**

From Jan. 1, 2000, to Dec. 31, 2007, 26 patients (with 29 involved hips) underwent liner exchange revision fixed with the original locking mechanism due to wear of CUHMWPE and/or osteolysis. The mean age was 53 ± 9 years at the time of the primary total hip arthroplasty (THA) and 64 ± 9 years at the revision. The exchanged liners (Marathon, Depuy) were made of HCLPE. Annual X-rays were used to measure linear wear and osteolysis. The annual linear penetration was measured using PolyWare® software (Draftware Inc.). Annual Harris Hip Scores(HSS) were recorded.

**Results:**

The mean follow-up time between the primary and revision THAs was 11 ± 2 years and 8 ± 2 years after revision. The mean Harris Hip Score(HHS) before primary THA, 1 year after primary THA, before revision and 1 year after revision was 43±5, 85±5, 71±6, 83±7 individually. The mean penetration of the CUHMWPE and HCLPE liners occurring in the first year were 0.44 ± 0.28 mm and 0.38 ± 0.14 mm, respectively (p = 0.211). The mean annual linear penetration of CUHMWPE and HCLPE from the second year onward were 0.29±0.09 mm and 0.08 ± 0.03 mm respectively (p <0.01). All THAs with CUHMWPE showed osteolysis on acetabular and/or femoral side before revision. No HCLPE liner showed osteolysis at the last follow-up. Conclusion: The CUHMWPE liner had a significantly higher wear rate than did the HCLPE liner. The HCLPE liner showed a satisfactory liner penetration rate after revision with isolated liner exchange fixed with the original locking mechanism.

## Introduction

Total hip arthroplasty (THA) is the most effective treatment method for advanced hip diseases. Polyethylene is commonly used as a bearing surface. Wear particles from polyethylene implants play an important role in the development of periprosthetic osteolysis, which can lead to prosthetic failure. Excessive wear and/or severe osteolysis can require revision, even if there is no pain [1]. Different types of modifications, including carbon-reinforced, highly crystalline, acetylene cross-linked, and high cross-linked, have been undertaken to improve the clinical performance of conventional ultra-high molecular weight polyethylene (CUHMWPE) since it was first used by John Charnley in 1962 [2]. Various of highly cross-linked polyethylene(HCLPE) liners (such as Marathon, Depuy; X3, Stryker; Longevity, Zimmer; XLPE, Smith and Nephew; and Acrom XL, Biomet) have been used extensively to decrease wear, osteolysis and related implant failures in THA. Studies[3–5] have also shown that HCLPE has much less wear and osteolysis than does CUHMWPE. But long-term result of HCLPE in revision THA is unknown.

In revision THA, isolated liner exchange is a common surgical intervention when the acetabular component remains well fixed. The intervention has several advantages, such as decreased morbidity and preservation of pelvic bone stock. Either the original locking mechanism or cement may be used to fix the revision liner into the retention cup. Although several follow up [6–9] about revisions with cemented liners shows good results theses years, the original locking mechanism may be preferred if it is available. But clinical data on the results after revision with isolated polyethylene liner exchange into a well-fixed, metal-backed shell with the original locking mechanism are limited.

It is important to determine polyethylene wear and osteolysis accurately in THA follow-up. Several methods, including manual methods [10, 11] and two- or three-dimensional computer-assisted techniques, such as MAXIMA [12], EBRA [13], PolyWare [14, 15], HAS [16], and radiostereometric analysis (RSA)[17, 18], have been developed since Charnley first reported on the subject [19]. RSA is the most accurate method. However, it requires the implantation of tantalum beads first, which limits the type and number of patients who can be studied. Computer-assisted techniques are popularly used. To improve the accuracy, precision and comparability, patients with same type of liner, prosthesis and femoral head diameter are selected besides standard clear AP radiographs of the pelvis without rotation.

In the present study, revisions with isolated HCLPE liner (all with Marathon, Depuy) exchange that had the same hip ball diameter (28 mm) and were fixed with the original locking mechanism were followed up. We wanted to compare the linear penetration rates of polyethylene before (CUHMWPE) and after (HCLPE) isolated liner exchange in patients who underwent THA revision. The reuse of locking mechanism were also studied.

## Materials and Methods

A retrospective waiver was obtained from the Ethics Committee of Shuguang Hospital. Authors had no access to identifying information and written informed consent was obtained from participants.

A total of 60 patients underwent hip revision with isolated liner exchange between Jan. 1, 2000, and Dec. 31, 2007, in our hospital. In all cases, the revision was due to polyethylene wear and osteolysis with a well-fixed acetabular component. We excluded patients whose exchanged liners were not Marathon (with an inner diameter of 28 mm) or were fixed with cement. After exclusions, 26 patients (13 men and 13 women with 29 involved hips) remained who underwent isolated liner exchange revision fixed with the original locking mechanism. Their case histories and X-rays were reviewed.

In all cases, the primary THA was performed via a posterolateral approach between Apr. 17, 1989, and Sep. 1, 1994. Etiologies for primary THA were as follows: avascular necrosis (23 hips), osteoarthritis (4 hips), and ankylosing spondylitis (2 hips). On the acetabulum side, a cementless, titanium, porous-coated shell (Duroloc®, DePuy) with a CUHMWPE liner (Enduron®, Depuy) was used with a press-fit technique. For the femoral component, a cementless, tapered, proximally titanium fiber metal and hydroxyapatite-coated stem (AML®, Depuy) was used. All heads that made of cobalt-chromium alloy were 28 mm in diameter.

The revisions were performed with the same approach by the same surgeon team. The stability of the metal shell was confirmed by both a preoperative X-ray and an intraoperative assessment. If necessary, careful debridement and artificial bone (Tricaleium phosphate, Triosite®, Zimmer) grafting of osteolytic lesions were performed through screw holes. Only one liner type, the HCLPE liner (Marathon, with an inner diameter of 28 mm), was used and was fixed with the original locking mechanism.

Clinical and radiographic evaluations were routinely performed preoperatively; at 6 weeks, 3 months, 6 months, and 1 year postoperatively; and annually thereafter. The Harris Hip Scoring System [20] was used to evaluate clinical outcomes. Anterior-posterior pelvic radiographs and lateral radiographs of the affected hips were obtained during hospitalization and at each follow-up.

All radiographs were digitized by a single independent observer. Inclination angles were measured. Polyethylene penetration and osteolysis were analyzed using computer-assisted PolyWare® software (Draftware Inc.) [14, 15]. The mean annual penetration rates[21, 22](i.e. polyethylene true wear and bedding-in which include creep and settling) were also calculated. Distinguish Periprosthetic osteolysis was defined as bone resorption indicated by a non-linear cystic lesion >5 mm wide[23].

Statistical analysis was performed using SPSS version 19.0 (SPSS, Chicago, Illinois). Harris Hip Scores, polyethylene wear before and after revision were compared using a paired t test.

## Results

The average age at the primary THA was 53±9 years. The mean diameter of the acetabular cup was 52±4 mm, the mean inclination angle was 50±5° and the mean anteversion was 20±4°. As a result of the primary THA, HHS was improved from 51±5 points (range, 37–61 points) preoperatively to 85±5 points (range, 70–95 points) 1 year postoperatively (p<0.01). (S1 File)

The mean age at revision was 63±9 years. The mean time interval between the primary and revision THA was 11±2 years. The reason for revision was polyethylene wear and osteolysis. All the patients with CUHMWPE liners showed osteolysis on the radiographs before revision. HHS was also improved from 71±6 points(range, 62–83) before revision to 81±7 points(range, 69–92) (p<0.01) 1year post revision. No one was lost to follow up.

**Fig 1:**
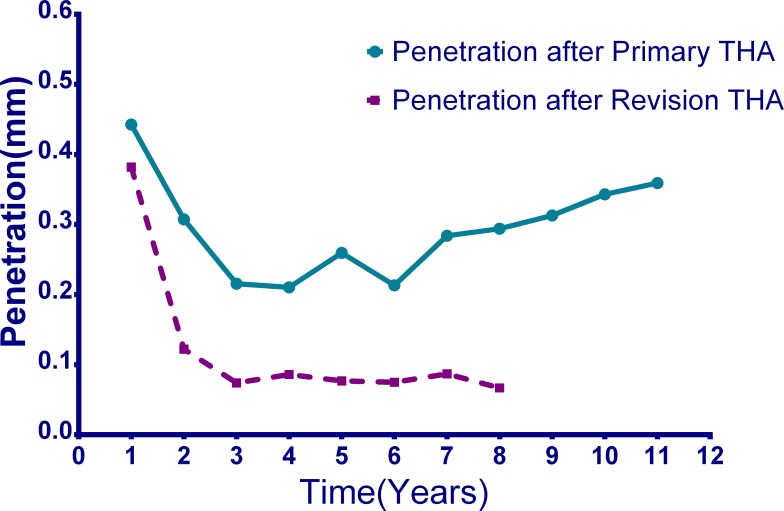
Penetration of CUHMWPE and HCLPE liners before and after revision.

The mean penetration of the CUHMWPE and HCLPE liners occurred in the first year were 0.44±0.28 mm (range, 0.16 to 1.1 mm) and 0.38±0.35 mm (range, 0.16 to 0.64 mm), respectively (p = 0.211). The mean annual linear penetration rates of the CUHMWPE and HCLPE from the second year onward were 0.29±0.09 mm per year and 0.08 ± 0.03 mm per year, respectively(p <0.01) (Fig 1). During a mean of 8 ±2 years follow-up after revision, no re-revision was done for any reason and no locking mechanical failure was indicated. One patient suffered a hip dislocation treated with closed reduction successfully approximate one month after revision. No periprosthetic infection occurred. No HCLPE patients showed osteolysis at the end of the follow-up period.

## Discussion

An accurate determination of polyethylene wear and osteolysis, and particularly any trends, can help us to understand the situation of the prosthesis and determine whether a revision should be performed. In vivo measurements of polyethylene wear are mainly based on comparisons of sequential pelvic radiographs. Penetration of polyethylene can be divided into true wear and bedding-in which includes creep and settling of liners. It is difficult to distinguish true wear from bedding-in[21, 22]. But our results showed that penetration in the first two years, especially in the first year was much higher than others because of bedding-in. It indicates that the bedding-in can be estimated by the penetration of the first two years minus average annual penetration after two years. It shows that although the wear of HCLPE is less than that of CUHMWPE, there seems no difference in bedding-in between them.

The risk of failure in patients who received a CUHMWPE liner at the time of the primary THA was 14.8 times higher than in patients who received an HCLPE liner because of high wear[24]. Although, fractures of HCLPE were also reported [25–27]. I our results, both CUHMWPE and HCLPE have a higher wear rate than the other average results [28] which were only 0.07–0.17 mm and 0.01–0.08 mm, respectively. This may be due to the fact that this specific group of patients, who all presented with osteolysis and were required revision surgery, had a higher wear rate than the general patient population. Therefore, they presented with higher wear rates than average. The inclination angles of these patients were also slightly higher, which may result in more wear. These findings also suggest that we should pay more attention to patients whose annual wear rates are more than 0.2 mm per year which increase the risk of osteolysis[29]. More frequent follow-up visits and more radiographic views are advised in these patients. If the polyethylene thickness is less than 3 mm, a revision should be performed, even in asymptomatic patients [30]. Regardless, after revision with an HCLPE exchange, the wear rate of HCLPE liners was still lower than 0.1 mm per year, which is regarded as the threshold for inducing osteolysis [2]. In addition, no osteolysis was found at the end of the follow-up with HCLPE liners. Another interesting phenomenon is that the penetration appears to increase in the final years. There may be more wear debris around the prosthesis and the femoral balls were in a more displaced position in the final years which promoted the wear. There were less patients followed-up in the later years which increased the bias as well.

Good results were shown about the reliability and validity of PolyWare method[31]. Ebramzadeh[32] also concluded that PolyWare had the lowest median error 0.1 mm. To increase the accuracy, some measures were adopted in this study. For example, Considering the possible nonconcentric position of the cemented liner, which could affect the measurement results, revisions with cemented liners were also excluded.

To address the polyethylene wear associated with osteolysis, isolated liner exchange is a common surgical intervention when the acetabular component remains well fixed [1, 24]. The results showed no locking mechanism failure, no acetabular loosening, and no osteolysis. Therefore, we suggest that the original locking mechanism should be used during isolated liner exchange revision if it is intact and if the corresponding HCLPE liner is available. Although short- and mid-term results of cementing the liner seem promising, long-term results are still unknown. It has been established that cementless acetabular components perform better than cement fixations over the long term [33].

The present study has several limitations. First, it was a retrospective, nonrandomized study, which has the potential for selection bias, and patients did not undergo surgery during the same time period. However, we included all (non-selected) patients who underwent an isolated polyethylene (Marathon) exchange revision fixed with the original locking mechanism. However, for this reason, the number of patients enrolled was very small. In addition, measurement error of polyethylene wear cannot be eliminated. We measured the same type of liner, prosthesis and femoral head diameter and compared the same patient’s data before and after revision to diminish the influence of measurement error.

## Conclusions

In conclusion, the CUHMWPE liner had a significantly higher wear rate compared with the HCLPE liner. The HCLPE liner showed a satisfactory liner penetration rate after isolated liner exchange. The original locking mechanism should be used if it is intact.

## Supporting Information

S1 File The demographic data, penetration and HHS of the patients. (SAV)
